# TRPML2 Mucolipin Channels Drive the Response of Glioma Stem Cells to Temozolomide and Affect the Overall Survival in Glioblastoma Patients

**DOI:** 10.3390/ijms232315356

**Published:** 2022-12-05

**Authors:** Maria Beatrice Morelli, Massimo Nabissi, Consuelo Amantini, Federica Maggi, Lucia Ricci-Vitiani, Roberto Pallini, Giorgio Santoni

**Affiliations:** 1 School of Pharmacy, University of Camerino, 62032 Camerino, Italy; 2 School of Biosciences and Veterinary Medicine, University of Camerino, 62032 Camerino, Italy; 3 Department of Hematology, Oncology and Molecular Medicine, Istituto Superiore di Sanità, 00161 Rome, Italy; 4 Institute of Neurosurgery, Gemelli University Polyclinic Foundation, Scientific Hospitalization and Care Institute (IRCCS), 00168 Rome, Italy; 5 Institute of Neurosurgery, School of Medicine, Catholic University, 00168 Rome, Italy

**Keywords:** glioblastoma, glioma stem cells, heterogeneity, ion channels, transient receptor potential mucolipin-2, temozolomide, overall survival, progression-free survival

## Abstract

The survival of patients with glioblastoma (GBM) is poor. The main cause is the presence of glioma stem cells (GSCs), exceptionally resistant to temozolomide (TMZ) treatment. This last may be related to the heterogeneous expression of ion channels, among them TRPML2. Its mRNA expression was evaluated in two different neural stem cell (NS/PC) lines and sixteen GBM stem-like cells by qRT-PCR. The response to TMZ was evaluated in undifferentiated or differentiated GSCs, and in TRPML2-induced or silenced GSCs. The relationship between TRPML2 expression and responsiveness to TMZ treatment was evaluated by MTT assay showing that increased TRPML2 mRNA levels are associated with resistance to TMZ. This research was deepened by qRT-PCR and western blot analysis. PI3K/AKT and JAK/STAT pathways as well as ABC and SLC drug transporters were involved. Finally, the relationship between TRPML2 expression and overall survival (OS) and progression-free survival (PFS) in patient-derived GSCs was evaluated by Kaplan–Meier analysis. The expression of TRPML2 mRNA correlates with worse OS and PFS in GBM patients. Thus, the expression of TRPML2 in GSCs influences the responsiveness to TMZ in vitro and affects OS and PFS in GBM patients.

## 1. Introduction

Glioblastoma (GBM) is the most aggressive malignant type of primary brain tumor with a high mortality rate. Generally, GBM has poor clinical outcomes and survival is rarely greater than 15 months after diagnosis [1,2]. It has been demonstrated that despite maximal resection of well-demarcated tumors combined with irradiation and chemotherapy, most tumors will recur due to resistance to therapy. The inter- and intra-heterogeneity of GBM, which inhibits proper treatment, is indicated to be at least partially responsible for poor patient outcomes [1,2]. Furthermore, a substantial body of evidence supports the existence of glioma-initiating or -propagating cells within GBMs. A subpopulation of tumorigenic cells exhibiting stem-like characteristics, the glioblastoma stem-like cells (GSCs), was shown to be responsible for relapse, resistance to therapy, and tumor maintenance. GSCs have been isolated from both human tumor tissues [3,4,5] and several glioma cell lines [4,6,7,8]. In addition to self-renewing and proliferating, GSCs can also initiate tumors upon secondary transplantation and generate progeny from multiple lineages [8]. As a result, GSCs assure the heterogeneity of GBM. In order to provide significant and personalized therapeutic strategies for patients with high-grade gliomas, it is therefore necessary to accurately identify different GSC subtypes in high-grade gliomas. Increasing evidence has revealed that several ion channels belonging to the transient receptor potential (TRP) family are heterogeneously expressed in different types of cancer stem cells (CSCs); however, information on GSCs is limited [9].

Among TRP, mucolipin (TRPML) channels are a group of three proteins (TRPML-1-3) mainly localized in endosomal and lysosomal compartments. Human TRPML2 encoded by the MCOLN2 gene is a Ca^2+^-permeable non-selective cation channel, which is inhibited by low extracytosolic pH and activated by phosphatidil-inositol 3,5 biphosphate [10,11]. The association between TRPML2 expression and cancer has been widely reported. Epigenetic methylation and a reduction of TRPML2 expression has been observed in pediatric acute lymphoblastic B-leukemia [12]. Moreover, TRPML2 is not detected or expressed only at low levels in lung adenocarcinoma and squamous cell carcinomas patients [13]. A significant correlation between the rs9929218 variant of cadherin-1 (CDH1), TRPML2 and colorectal cancer (CRC) susceptibility has been demonstrated. The analysis of the transcriptome in CRC showed a dramatic TRPML2 down-regulation [14] compared to normal tissue. An increased TRPML2 expression in HN31 oral cancer cells has been evidenced [15]. Moreover, TRPML2 overexpression promoting IL-1β/NF-κB-dependent prostate cancers proliferation, migration and invasion was associated with poor prognosis [16]. In breast cancer, TRPML2 expression was associated with clinical ER and HER2 phenotype, recurrence, metastasis and distinct survival patterns [17].

In regard to gliomas, we have previously found that TRPML2 silencing inhibits the viability, reduces the proliferation and induces caspase-3-dependent apoptosis in glioma cell lines [18]. Moreover, it inhibits the VEGF-A/Notch2 angiogenic pathway, whereas TRPML2 overexpression or its activation increases the VEGF-A/Notch2 expression. In addition, an increased invasion capability, epithelial–mesenchymal transition markers expression, and sensitivity to doxorubicin in silenced TRPML2 and a shorter OS in TRPML2 overexpressing GBM patients was reported [19]. Finally, overexpression of TRPML2 as well as the complete loss of this channel have been associated with worse OS and prognosis, whereas lower TRPML2 expression shows a protective effect in GBM patients [18,20]. In agreement, the Chinese Glioma Genome Atlas (CGGA) database reveals high TRPML2 expression in high-grade GBM, which correlates with shorter OS and worse prognosis, whereas favorable DFS is associated with lower levels of TRPML2; moreover, high TRPML2 levels are associated with 1p/19q non-codeletion and IDH-wild type status [21].

The aim of the present work is to determinate the expression of TRPML2 in 16 different GSC lines. Moreover, the relationship between TRPML2, TMZ response and resistance mechanisms, and the clinical relevance between the TRPML2 mRNA expression and the OS and PFS in GBM patients will be evaluated.

## 2. Results

### 2.1. TRPML2 mRNA Expression during Differentiation of Neural Stem/Progenitor Cells (NS/PCs)

**Figure 1:**
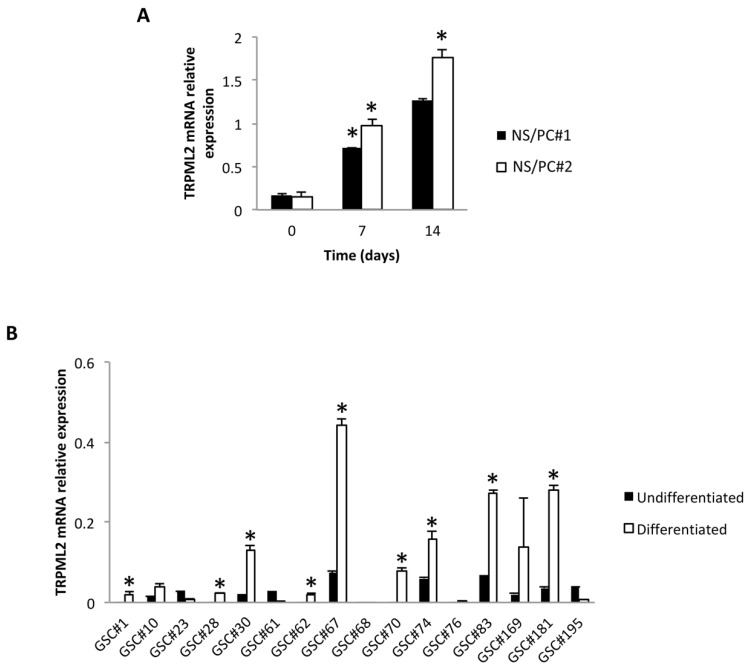
TRPML2 mRNA expression in neural stem/progenitor cells and glioma stem-like cells. (**A**) The relative TRPML2 mRNA expression in human neural stem/progenitor (NS/PC#1 and NS/PC#2) cells was evaluated by qRT-PCR during the differentiation. TRPML2 mRNA levels were normalized for GAPDH expression. Data are expressed as mean ± SD. * *p* < 0.01 vs. time 0. (**B**) The relative TRPML2 mRNA expression was evaluated by qRT-PCR in undifferentiated GSC lines and after 14 differentiation days. TRPML2 mRNA levels were normalized for GAPDH expression. Data are expressed as mean ± SD. * *p* < 0.05 vs. undifferentiated cells.

We previously reported that TRPML2 mRNA is more highly expressed in NS/PC than in normal brains (NHBs) and normal human astrocytes (NHAs) [18]. Here, by quantitative RT-PCR, we evaluated the TRPML2 expression during the differentiation of two different NS/PC (NS/PC#1 and NS/PC#2) lines. We found that TRPML2 mRNA levels strongly and progressively increase by a factor of 4.3 and 7.5 for NS/PC#1; or of 6.3 and 11.5 for NS/PC#2, at 7 and 14 days of differentiation, respectively, in 5% FBS (Figure 1A).

### 2.2. TRPML2 mRNA Expression in Undifferentiated (GSCs) and Differentiated (D-GSCs) Glioma Stem-like Cell Lines

Next, the expression of TRPML2 mRNA in 16 undifferentiated GSC lines (GSC#1#, GSC#10, GSC#23, GSC#28, GSC#30, GSC#61, GSC#62, GSC#67, GSC#68, GSC#70, GSC#74, GSC#76, GSC#83, GSC#169, GSC#181 and GSC#195) derived from GBM patients [22] (Table S1) was evaluated by quantitative RT-PCR (Figure 1B). We found that 6 out of 16 GSC lines (37.5%) were TRPML2 negative, and 10 out of 16 (62.5%) express different TRPML2 mRNA levels. Moreover, at 14 days, differentiation of these GSC lines induced TRPML2 ex-novo expression in 4 lines (GSC#1, GSC#28, GSC#62, GSC#70); GSC#68 and GSC#76 remained negative; GSC#10, GSC#30, GSC#67, GSC#74, GSC#83 and GSC#181 showed an increased TRPML2 mRNA expression; while a reduction of TRPML2 mRNA levels was evidenced in GSC#23, GSC#61 and GSC#195 lines, compared to the respective undifferentiated GSCs (Figure 1B).

The analysis of the TRPML2 mRNA expression in the GSC positive lines evidenced lower levels compared with the NS/PCs, suggesting that transformation of NS/PC in GSC can be correlated with TRPML2 reduction.

In addition, in contrast with NS/PCs where the TRPML2 mRNA expression progressively increases during the NS/PC differentiation, the differentiation of GSCs further increases the TRPML2 heterogeneity (12.5% negative, 25% ex novo induced; 18.7% reduced and 43.7% increased TRPML2 mRNA expression).

### 2.3. TMZ Resistance Is Associated with TRPML2 mRNA Expression in Undifferentiated and Differentiated GSCs Lines

**Figure 2:**
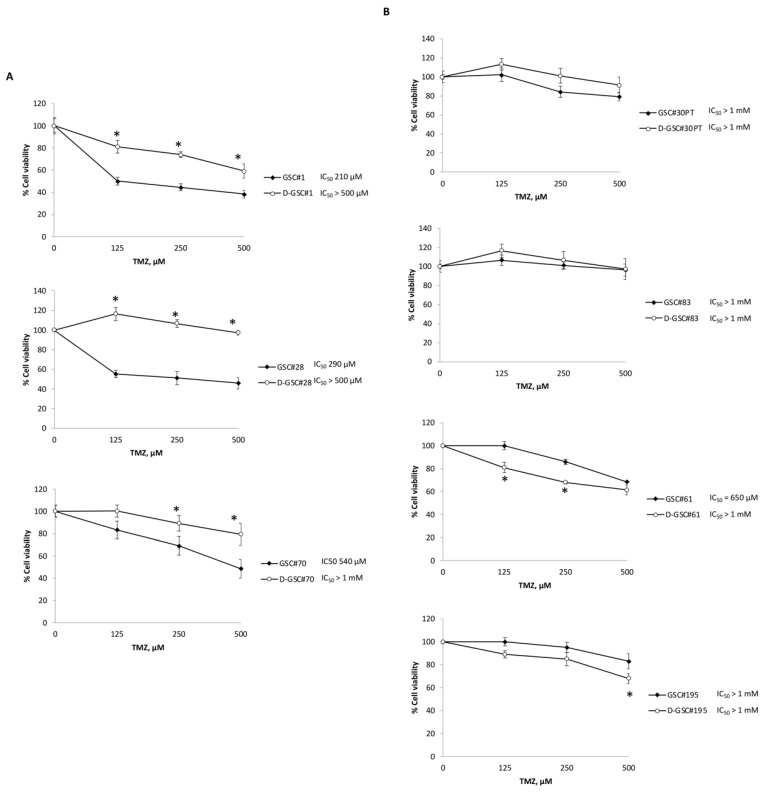
Effects of TMZ on growth of undifferentiated (GSC) and differentiated (D-GSC) cell lines. Cell growth was evaluated by 3-(4,5-dimethylthiazol-2-yl)-2,5-diphenyltetrazolium bromide (MTT) assay in TRPML2 negative GSC (**A**) and in TRPML2 positive GSC (**B**). Data shown are expressed as mean ± SE of three separate experiments. * *p* < 0.05 vs. GSC.

The 14 days’ GSC differentiation is associated with induction (GSC#1) or increase (GSC#28 and GSC#70) of TRPML2 mRNA expression. Thus, the effect of TMZ treatment (125, 250 and 500 μM) was evaluated by an MTT assay in undifferentiated and differentiated D-GSC#1, D-GSC#28 and D-GSC#70 (Figure 2). We found that differentiation of GSC at 14 days, which increases the TRPML2 mRNA expression, impairs the sensitivity to TMZ treatment (GSC#1; IC50 > 500 vs. 210, GSC#28 > 500 vs. 290 and GSC#70 > 1000 vs. 540 μM). No significant changes in TMZ resistance were evidenced in undifferentiated TRPML2-positive D-GSC#30, D-GSC#83, D-GSC#61 and D-GSC#195 compared to undifferentiated cells.

Thus, changes (whether increase or reduction) in the TRPML2 mRNA expression, happening during GSC differentiation, parallel the increase of TMZ-resistance in TMZ-sensitive lines. On the other hand, TRPML2-positive GSCs were resistant to TMZ treatment and no changes in TMZ sensitivity were evidenced upon differentiation. Overall, these results suggest the existence of a relationship between TRPML2 expression and TMZ response in GSCs.

### 2.4. Changes in the TRPML2 mRNA Expression Result in Modulation of the Responsivity of GSC Lines to TMZ Treatment

**Figure 3:**
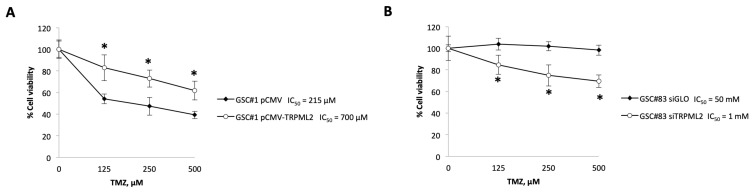
Effects of TMZ on growth of GSC#1 and GSC#83. Cell growth after 48 TMZ treatment was evaluated by MTT assay in (**A**) TRPML2 negative GSC#1 (pCMV) and in GSC#1 pCMV-TRPML2, and (**B**) TRPML2 positive GSC#83 (siGLO) and in GSC#83 siTRPML2. Data shown are expressed as mean ± SE of three separate experiments. * *p* < 0.05 vs. control cells (GSC#1 pCMV and GSC#83 siGLO).

To further address the relationship between the TRPML2 mRNA expression and drug resistance or sensitivity to TMZ treatment, we transfected pCMV-TRPML2 in the TRPML2 negative GSC#1 line (pCMV-TRPML2 proneural GSC#1) and silenced by RNA interference the TRPML2 mRNA expression in TRPML2-positive GSC#83 line (siTRPML2 GSC#83 line) (Figure S1). The TRPML2 mRNA and protein levels after transfection were confirmed by qPCR and western blot analysis (Figure S1). Cell viability results revealed that TRPML2 overexpression conferred increased resistance (IC_50_ 700 μM) to TMZ compared with pCMV GSC#1 control cells (IC_50_ 215 μM) (Figure 3A). In GSC#83 TRPML2, silencing reduced the TMZ-resistance even though the IC_50_ value remains high (IC_50_ 1 mM in siTRPML2 vs. 50 mM in siGLO) (Figure 3B). No major differences in the TMZ cytotoxic effects were evidenced comparing untreated vs. pCMV or siGLO control GSC lines (data not shown).

**Figure 4:**
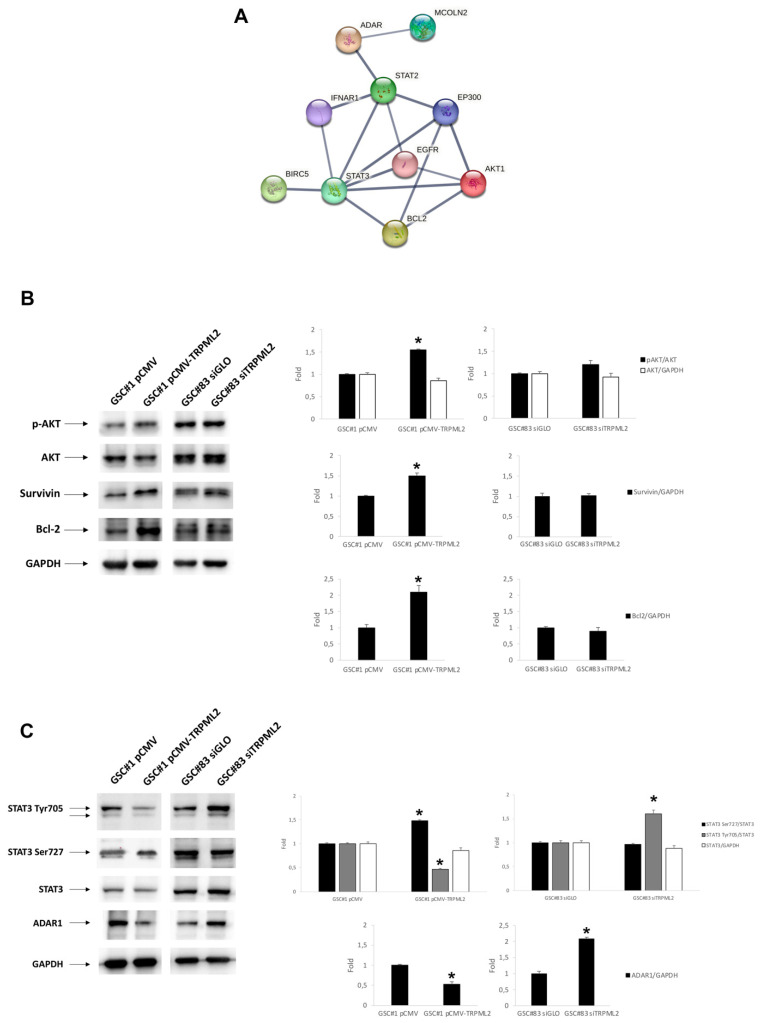
Key candidate proteins in TMZ resistance related to TRPML2 expression. (**A**) The protein–protein interaction network derived from STRING connects the selected markers (PPI enrichment, *p* value = 0.01). (**B**) Total cell lysates were subjected to western blot analysis to detect the expression levels of AKT, pAKT, STAT3, pSTAT3-Tyr705, pSTAT3-Ser727, ADAR1, Survivin (encoded by Birc5) and Bcl2 using the specific antibodies as indicated, with GAPDH as the loading control. Representative images are shown from one of three independent experiments, which produced similar results. Densitometric analysis assessed the relative protein expression levels in three independent experiments. The results are the mean ± SD. Akt, STAT3, ADAR1, Survivin and Bcl2 densitometry values were normalized to GAPDH. (**C**) The pSTAT3-Tyr705 and pSTAT3-Ser727 protein levels were determined with respect to STAT3 levels. The pAkt protein levels were determined with respect to Akt levels * *p* < 0.05 vs. control cells.

Given that different sensitivity to TMZ and also the different TRPML2 expression was found in the analyzed cell lines, we assessed whether specific molecular pathways related to TMZ resistance [23] could be influenced by TRPML2 expression. Using the STRING online database (https://string-db.org (accessed on 8 November 2022)), a Protein–Protein Interaction (PPI) network was constructed (Figure 4A). The analysis showed that both PI3K/Akt and JAK/STAT pathways can be modulated. Thus, to analyze that effect in more detail, the key protein expression levels were evaluated by western blot. Data showed that both pathways are regulated by the different expressions of TRPML2. There was a significant increase in Akt signaling in the pCMV-TRPML2 GSC#1 line with respect to control cells (Figure 4B). Immunoblots confirmed that phosphorylation levels of Akt (pAKT) increase even though the total Akt form remains unchanged. In addition, the Akt-mediated anti-apoptotic target Bcl-2 and BIRC5 are upregulated in GSC#1 transfected cells. Moreover, the expression level of STAT3 and its phosphorylation status were investigated in control and transfected cells (Figure 4C). The levels of STAT3 were unaffected, while the levels pSTAT-Ser727 were increased in the pCMV-TRPML2 GSC#1 cells as compared with the control cells. On the other hand, the level of pSTAT3-Tyr705 was decreased. In siTRPML2 GSC#83 cells, we detected only an increase in STAT3-Tyr705. TRPML2-related proteins in PPI include also ADAR1, a dsRNA-editing enzyme. As shown in Figure 4C, ADAR1 expression is inversely proportional to TRPML2 levels in both GSC#1 and GSC#83 cell lines.

### 2.5. Drug-Transporter Gene Profile in TRPML2-Negative GSC#1 and TRPML2-Positive GSC#83 Lines

**Figure 5:**
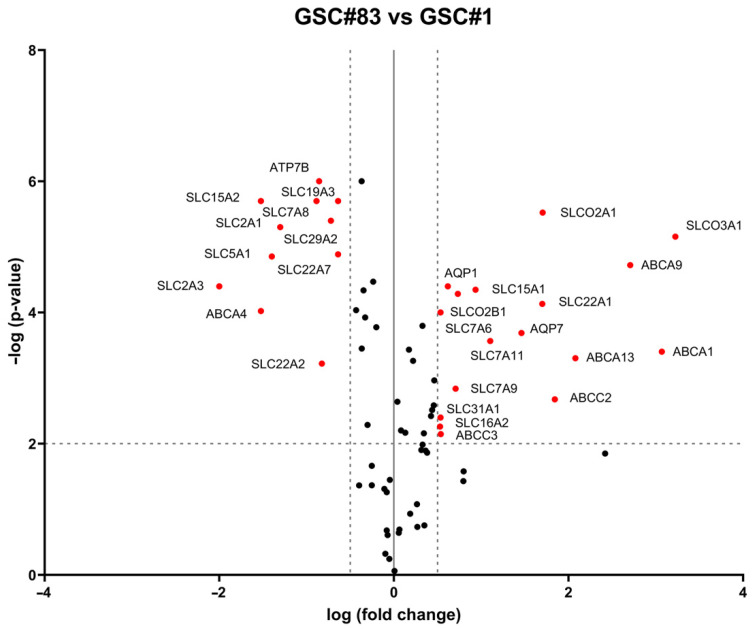
Differential expression analysis between GSC#83 and GSC#1 cell lines. Volcano plot illustrates the relative expression levels for each gene depicted as log 10 (n-fold) plotted against –log 10 (*p*-value) between GSC#83 and GSC#1. Horizontal bar at y = 2 represents a significance level of *p* =0.01; vertical bars at x = ±0.5 represent the fold change threshold (genes in black did not reach significance). The red plus signs represent upregulated or downregulated differentially expressed genes; the black circles represent non-differentially expressed genes. Genes not expressed in both cell lines were excluded from the graph. ATP-binding cassette, sub-family A (ABC1), member 1 (ABCA1); ABC1 member 4 (ABCA4); ABC1 member 9 (ABCA9); CFTR/MRP member 2 (ABCC2); CFTR/MRP member 3 (ABCC3); Aquaporin 1 (AQP1); Aquaporin 7 (AQP7); ATPase, Cu++ transporting, beta polypeptide (ATP7B); solute carrier family 15 (oligopeptide transporter), member 1 (SLC15A1); solute carrier family 15 (H+/peptide transporter), member 2 (SLC15A2); solute carrier family 16, member 2 (monocarboxylic acid transporter 8) (SLC16A2); solute carrier family 19, member 3 (SLC19A3); solute carrier family 22 (organic cation transporter), member 1 (SLC22A1); solute carrier family 22 (organic cation transporter), member 2 (SLC22A2); solute carrier family 22 (organic anion transporter), member 7 (SLC22A7); solute carrier family 29 (nucleoside transporters), member 2 (SLC29A2); solute carrier family 2 (facilitated glucose transporter), member 1 (SLC2A1); solute carrier family 2 (facilitated glucose transporter), member 3 (SLC2A3); solute carrier family 5 (sodium/glucose cotransporter), member 1 (SLC5A1); solute carrier family 7 (amino acid transporter light chain, L system), member 8 (SLC7A8); solute carrier family 7 (glycoprotein-associated amino acid transporter light chain, bo, +system), member 9 (SLC7A9); solute carrier family 7 (glycoprotein-associated amino acid transporter light chain, bo, +system), member 11 (SLC7A11); solute carrier organic anion transporter family, member 2A1 (SLCO2A1); solute carrier organic anion transporter family, member 2B1 (SLCO2B1); solute carrier organic anion transporter family, member 3A1 (SLCO3A1).

Drug membrane transporters from ATP-binding cassette (ABC) sub-family A, B, C and D, solute carrier transporter (SLC) and aquaporin (AQP) families play a main role in the resistance of GBM stem cells to TMZ and other anti-neoplastic agents employed in the care of glioma tumors [24]. Thus, the expression of ABC, SLC and AQP drug transporter genes was evaluated in TRPML2-negative GSC#1 and TRPML2-positive GSC#83 lines by RT2 Profiler PCR Array (Table S2). In total, 28 DEGs were identified. The volcano plot showed the distribution of DEGs (Figure 5). We found that ATP7B, SCL15A2, SCL7A8, SLC19A3, SCL2A1, SLC29A2, SLC5A1, SLC22A7, SLC2A3, ABCA4, and SLC22A2 are more expressed in GSC#1; while SLCO2A1, SLCO3A1, ABCA9, AQP1, SLC15A1, SLCO2B1, SLC22A1, SLC7A6, AQP7, SLC7A11, ABCA13, ABCA1, ABCC2, SLC7A9, SLC31A1, SLC16A2, and ABCC3 are more expressed in GSC#83 compared to GSC#1. 

### 2.6. Drug Transporter Genes Correlated to TRPML2 Expression in GSC Lines 

To deepen the study of drug transporters and their relationship with the TRPML2 channel modulation, we evaluated the drug transporter gene expression in pCMV-TRPML2 overexpressed GSC#1 and in siTRPML2 GSC#83 lines, with respect to their control cells (Table S3).

**Figure 6:**
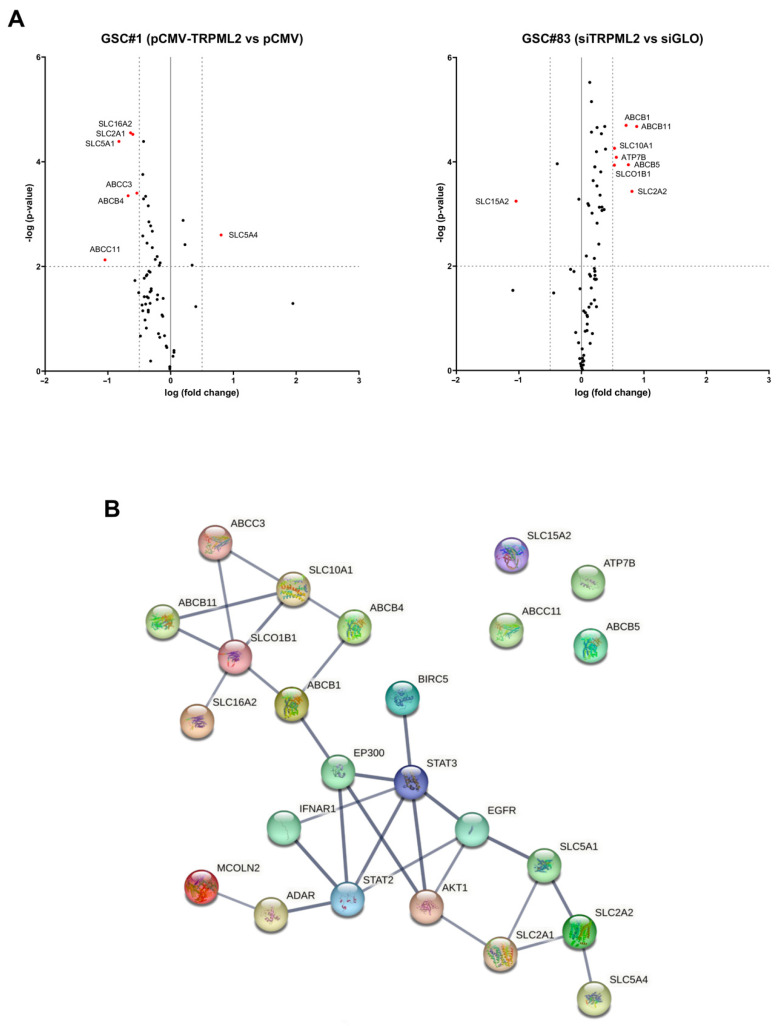
Differential expression analysis in GSC#83 and GSC#1 cell lines related to the degree of expression of TRPML2. (**A**) Volcano plot illustrates the relative expression levels for each gene depicted as log 10 (n-fold) plotted against –log 10 (*p*-value) between (left) pCMV GSC#1 and pCMV-TRPML2 #1, and (right) siTRPML2 GSC#83 and siGLO GSC#83. Horizontal bar at y = 2 represents a significance level of *p* = 0.01; vertical bars at x = 0.5 represent the fold change threshold. The red plus signs represent upregulated or downregulated differentially expressed genes, and the black circles represent non-differentially expressed genes. ATP-binding cassette, sub-family B (MDR/TAP), member 1 (ABCB1); ATP-binding cassette, sub-family B (MDR/TAP), member 11 (ABCB11); MDR/TAP member 4 (ABCB4); MDR/TAP member 5 (ABCB5); CFTR/MRP member 3 (ABCC3); CFTR/MRP member 11 (ABCC11); ATPase, Cu++ transporting, beta polypeptide (ATP7B); solute carrier family 10 (sodium/bile acid cotransporter family), member 1 (SLC10A1); solute carrier family 15 (H+/peptide transporter), member 2 (SLC15A2); solute carrier family 16, member 2 (monocarboxylic acid transporter 8) (SLC16A2); solute carrier family 2 (facilitated glucose transporter), member 1 (SLC2A1); solute carrier family 2 (facilitated glucose transporter), member 2 (SLC2A2); solute carrier family 5 (sodium/glucose cotransporter), member 1 (SLC5A1); solute carrier family 5 (low affinity glucose cotransporter), member 4 (SLC5A4); solute carrier organic anion transporter family, member 1B1 (SLCO1B1). (**B**) String analysis for protein–protein interactions is done on differently expressed genes, TRPML2 and key proteins involved in TMZ resistance (*p* value = 2.07 × 10^−7^).

The analysis of genes belonging to the ABC, SLC and AQP families evidenced that among the modified ones, there are six genes whose expression is significantly modulated in the GSC#1 transfected model and seven genes in the GSC#83 silenced model (Figure 6A). In particular, SLC16A2, SLC2A1, SLC5A1, ABCC3, ABCB4, and ABCC11 expression decreases and SLC5A4 increases in pCMV-TRPML2 GSC#1 with respect to pCMV cells. SLC15A2 decreases and ABCB1, ABCB11, SLC10A1, ATP7B, ABCB5, SLCO1B1, and SLC2A2 increase in siTRPML2 GSC#83 with respect to siGLO cells. In general, an opposite trend of gene modulation is visible.

Based on the information in the STRING protein query from public databases, we made the PPI network of the identified 13 DEGs, TRPML2 and proteins involved in TMZ resistance modulated in our models on the basis of the western blot analysis (Figure 4). All proteins, except for SCL15A2, ATP7B, ABCC11 and ABCB5, are interconnected, which supports the hypothesis of a link between TRPML2 and resistance to TMZ (Figure 6B).

### 2.7. Correlation between the Tumor and Clinical Characteristics and OS and PFS 

**Figure 7:**
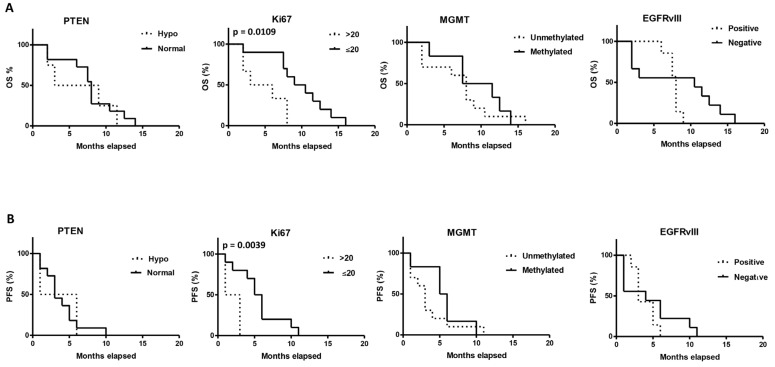
Survival analysis by Kaplan–Meier curves and long-rank (Mantel–Cox) test. The difference of OS (**A**) and PFS (**B**) in GBM patients stratified on the basis of PTEN status (hypophosphorylated/normal), Ki67 positivity, MGMT methylation status (unmethylated/methylated), and EGFRvIII positive/negative.

We evaluated the correlation between the OS or PFS and the clinico-pathological parameters investigated at the time of diagnosis at the Institute of Neurosurgery, Catholic University School of Medicine, in Rome (Italy) (Figure 7, Table S1). Age (<60 vs. >60 years), sex (male vs. female), tumor localization (temporal vs. parietal vs. frontal), Ki67 (low vs. high), PTEN (normal vs. hypophosphorylated), MGMT (unmethylated vs. methylated), EGFRvIII (negative vs. positive) have been taken into consideration. By Kaplan–Meyer analysis, a positive correlation with higher OS and PFS was evidenced for Ki67 low ≤ 20 vs. Ki67 high > 20 (OS: 9.75 vs. 4.5 months, *p* = 0.0109; PFS: 5.5 vs. 2.0 months, *p* = 0.0039). No positive correlation was evidenced for OS or PFS with age, sex, and tumor localization (data not shown), PTEN, MGMT and EGFRvIII.

### 2.8. The TRPML2 mRNA Expression Correlates with Poor OS and PFS in GBM Stem Cell-Derived Patients 

**Figure 8:**
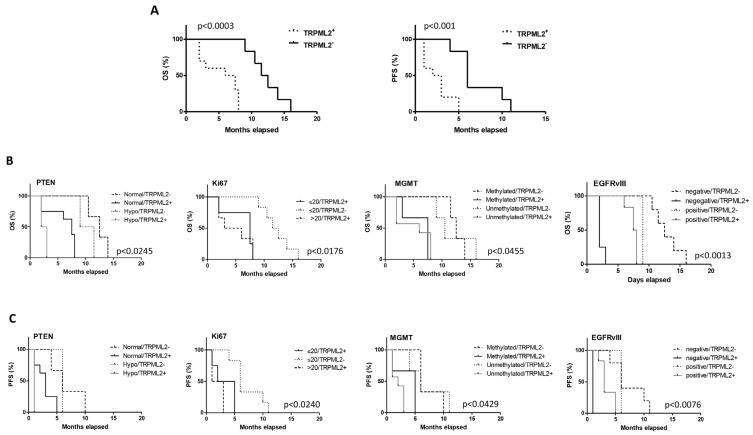
Survival analysis by Kaplan–Meier curves and long-rank (Mantel–Cox) test. Stratification of GBM patients based negative or positive TRPML2 expression (**A**), PTEN hypophosphorylated/normal, Ki67 positivity, MGMT methylation status, EGFRvIII positive/negative (**B**,**C**).

The expression profiling of several TRP ion channels, including the TRPC1, TRPM4 TRPML1 and TRPML2 genes, has been reported to predict the clinical outcome in cancer patients [19,25,26]. Thus, the correlation between TRPML2 mRNA expression and OS and PFS was evaluated. Statistical analysis by Kaplan–Meier reveals a significant difference in the OS (*p* = 0.0003) and PFS (*p* = 0.0062) in TRPML2-positive (n = 10/16) vs. TRPML2 negative (n = 6/16) patients (OS: TRPML2-positive 6.75 vs. TRPML2-negative 12.0 months; PFS: TRPML2-positive 2.5 vs. 6.0 months) (Figure 8A). 

Moreover, the OS and PFS in methylated or unmethylated MGMT, high > 20 vs. low ≤ 20 Ki67, PTEN normal vs. hypophosphorylated and EGFRvIII negative vs. positive in TRPML2 positive or negative GSCs were evaluated (Figure 7B, Table S4). Regarding methylated MGMT, TRPML2-negative compared to TRPML2-positive showed longer OS (12.5 vs. 7.5 months, *p* = 0.0295) and PFS (6 vs. 5months, *p* = 0.0295); regarding unmethylated MGMT, TRPML2-positive patients showed lower OS (6 vs. 10.5 months, *p* = 0.0094) and PFS (2 vs. 6 months, *p* = 0.0094) compared to TRPML2-negative patients-derived GSCs. 

A strict relationship between EGFRvIII and TRPML2 expression was also observed in GSCs. Thus, longer OS (7.75 vs. 2 months, *p* = 0.0013) and PFS (3.0 vs. 1.0 months, *p* = 0.0027) were evidenced in EGFRvIII-positive vs. EGFRvIII-negative TRPML2-positive patients; by contrast, longer OS were observed in EGFRvIII-negative vs. EGFRvIII-positive TRPML2-negative patients (12.5 vs. 9.0 months, *p* = 0.0253). No differences in PFS were observed comparing TRPML2-negative EGFRvIII-positive vs. TRPML2-negative EGFRvIII-negative patients. In addition, TRPML2-negative EGFRvIII-negative patients showed longer OS (12.5 vs. 2.0 months, *p* = 0.0027) and PFS (6.0 vs. 1.0 months, *p* = 0.0047) with respect to TRPML2-positive EGFRvIII-negative patients.

Regarding Ki67, low Ki67 and negative TRPML2 expression correlate with longer OS and PFS compared to Ki67low/TRPML2positive (OS: 12.0 vs. 7.5 months, *p* = 0.0013; PFS: 6.0 vs. 3.5 months, *p* = 0.0140) and Ki67 high/TRPML2 positive (OS: 12.0 vs. 4.5 months, *p* = 0.0007; PFS: 6.0 vs. 2.0 months, *p* = 0.0009).

Finally, comparing normal PTEN in TRPML2-positive vs. TRPML2-negative patients, a shorter OS (7.5 vs. 12.5 months, *p* = 0.0089) and PFS (3.0 vs. 6.0 months, *p* = 0.043) was evidenced. 

## 3. Discussion

The glioblastoma cell population is heterogeneous, with tumor-differentiated cells coexisting with subpopulations displaying stem cell characteristics. It is thought that GSCs derived from the normal NS/PC compartment [27] are responsible for recurrence and clinical relapse of glioblastoma [28,29,30]. Given that pharmacological modulation of the TRP ion channel activity in cancer cells is linked to their sensitivity to chemotherapeutic drugs [31], our goal was to examine the TRPML2 expression in GSCs and its relationship to resistance to TMZ, the standard chemotherapy for newly diagnosed GBM since 2005 and the subsequent use of the Stupp regimen [32]. The treatment of GBM with TMZ is not successful in over 50% of patient cases; however, there are few predictive markers beyond MGMT status for GBM patients treated with TMZ [23].

Using two NS/PCs lines and 16 GSC lines, we evidenced a lower expression of TRPML2 in GCSs with respect to normal cells and, above all, a different regulation during the differentiation process. While the normal cells show an increase in the channel expression levels, the GSCs do not seem to follow a single trend, but display an aberrant multipotent differentiation along neuronal, astroglial and oligodendroglial cell maturation [33]. We then exploited the different regulation of TRPML2 during differentiation in different GSC lines to assess whether there was a correlation with TMZ resistance. In those GSC lines where TRPML2 is expressed at higher levels, the resistance to TMZ treatment is even more marked. Instead, the TRPML2 down-regulation did not significantly change drug response. Thus, these data support the idea of a role for TRPML2 in tumor transformation and also in TMZ resistance. Moreover, TRPML2 overexpressed and silenced models were used. The expression of TRPML2 in TRPML2-negative GSC#1 increased the resistance of GSC to TMZ treatment; by contrast, silencing of TRPML2 mRNA in TRPML2-positive GSC#83 cells increases the sensitivity. Therefore, in GSCs, the more TRPML2 is expressed, the more resistant the cells are to TMZ. 

Several mechanisms of TMZ resistance have been described to-date [23]. With STRING analysis, we assessed which of the key molecular pathways could be connected with the TRPML2 channel, and western blot analysis confirmed that PI3K/Akt and JAK/STAT pathways are modulated in our models. Dysregulation of the PI3K/Akt pathway occurs in up to 88% of GBM tumors and Akt, also known as protein kinase B, is a serine/threonine kinase that plays a crucial role in promoting chemoresistance in GBM cells [34]. Several downstream targets of Akt have been found to be implicated in specific mechanisms of TMZ resistance, including apoptotic regulators such as Bcl-2. The balance between the expression level of anti-apoptotic proteins and pro-apoptotic proteins determines the fate of cancer cells and the development of chemotherapeutic resistance [35]. Additionally, Survivin, a member of the inhibitor of apoptosis family [36], confers TMZ resistance by blocking the effect of TMZ-induced apoptosis. Furthermore, it has been demonstrated that TMZ sensitivity can be increased by targeting the Survivin gene [37]. Signaling through the JAK/STAT pathway stimulates the stemness of glial cells. Specifically, activating STAT3 is known to affect the transition from proneural to mesenchymal GBM type [38,39] that is associated with more aggressive and multitherapy-resistant features [40]. In support of this, mesenchymal GSC#83 cells express more STAT3 to begin with, have more of it activated and are more resistant to TMZ treatment than proneural GSC#1 cells. However, STAT3 can be differentially activated to regulate cancer cell phenotype and control their fate. The phosphorylation of a serine at position 727 in the absence of Tyr705 phosphorylation correlates with the survival of neural stem cells [41] and it is also characteristic of TMZ-resistant glioma cells [42]. These reports are in agreement with our result in that the expression of pSTAT3-Ser727 was increased in pCMV-TRPML2 GSC#1 cells that are more resistant to TMZ as compared with the control cells.

The STRING analysis also showed a correlation between TRPML2 and ADAR1. This protein is a dsRNA-editing enzyme that catalyzes the conversion of adenosine to inosine. Even though many editing sites in the micro-RNA transcriptome have been discovered [43,44,45], the overall biological significance of ADARs is still largely unknown [46]. In most cases, increased ADAR promotes cancer generation and progression; while in a few cancers, low expression and/or activity of ADAR mediates cancer phenotypes [47,48]. In melanoma cells, an impairment of ADAR1 activity promotes cancer cell growth and metastasis [48]. In breast cancer, the migration and invasion ability are related to ADAR1 expression [47]. In GBM, Jiang et al. demonstrated that ADAR1 contributes to GSC self-renewal [49]. Furthermore, ADAR1 was shown to be involved in the impairment of TMZ resistance in glioma stabilizing glutaminase 2 (GLS2) mRNA, involved in the ferroptosis pathway by lipid metabolism [50]. Our work for the first time relates TRPML2 mediated regulation of ADAR1 expression to drug resistance, by which the lower the expression of ADAR1, the greater the resistance of cells.

Drug-resistance can also be attributed to an altered expression of ATP-dependent drug efflux pumps and drug efflux mediated by ABC and SLC transporters leading to a decreased cellular accumulation of anticancer drugs. This is considered a major drawback of currently applied chemotherapy regimens, and abnormal low expression of drug transporters in GBMs has been associated with tumor malignancy. Dysregulation in the endolysosome compartment is involved in mediating drug resistance [51] and several drug resistance transporters have been found in the endolysosomal system [52]. Since drug efflux capacity has been associated with stem cells derived from neoplastic tissues [53], we analyzed its involvement in TRPML2-mediated TMZ resistance in GSCs. Data demonstrated that changes in the TRPML2 expression markedly affected drug transporter gene expression in GSCs. Thus, ABCC3/MRP3, ABCB4/MDR3, ABCC11/MRP8, SLC16A2 SLC2A1/GLUT-1, and SLC5A1 were downregulated, and SLC5A4 was upregulated by TRPML2 gene transfection in GSC#1 cells; on the other hand, silencing of TRPML2 in GSC#83 induced ABCB1/MDR1/P-gp, ABCB5/MDR5, ABCB11/MRP11, SLCO1B1/OATP1B1, SLC2A2/GLUT-2, and SLC10A1/NTCP, and reduced SLC15A2 mRNA expression. Among these transporters, according to [33], ABCB4 significantly correlates with the CD133 stem cell marker expression and poor OS in GBM patients with the CD133 + GSCs, contributing to TMZ-resistance by exhibiting reduced responsiveness to TMZ, compared to CD133 − GSCs. ABCB1, which encodes for Pgp, is highly expressed in GSCs but TMZ treatment reduces its transcriptional activation [54]. Moreover, ABCC3 can be regulated by TMZ administration [55]. Instead, ABCC11 and SLC10A1 has no documented interaction with TMZ. SLC5A1, which encodes a member of the sodium-dependent glucose transporter, promotes ferroptosis [56], an alternative pathway of cell death that can be targeted to reverse TMZ resistance in glioma [57]. Furthermore, the function of influx transporters, in particular the solute carriers (SLC) in cancer cells, has been recently reassessed regarding cancer therapy. Indeed, the SLC transporters also serve as the uptake mediators of essential nutrients for tumor growth and survival [58]. Given their role as glucose transporters and the importance of a highly efficient glucose uptake for brain tumor-initiating cell growth, these proteins play a significant role in glioblastoma survival [59]. In light of their well-established contribution in promoting metabolism, elimination and detoxification of chemotherapeutic drugs, the regulation of some of the analyzed genes may seem a little surprising given their well-established role in reducing therapeutic effectiveness and treatment failure. Nevertheless, emerging research has demonstrated that drug carriers can impart either drug resistance or drug sensitivity, depending on the context. Additionally, rather than considering the expression of each single protein, in the context of a drug resistant or sensitive phenotype, it is also critical to consider the ratio of efflux (ABC) to influx (SLC) transporters [60].

In conclusion, the limited effectiveness of TMZ in GBM can be correlated with deficits in apoptosis induction, activation of multiple signaling pathways, and extrusion of drugs through cell membrane. However, although new therapeutic targets have been identified, the overall survival of GBM patients remains dismal due to tumor recurrence followed by chemoresistance. Increasing evidence has identified TRPML2 as a potential biomarker [18,19]. Herein, Kaplan–Meier analysis, supported by our in vitro models, correlates TRPML2-positive expression in patients’ derived GSCs with poor OS and PFS. 

It is well demonstrated that the sensitivity to TMZ is significantly associated with MGMT methylation status. The work by [61] shows that GBM patients harboring methylated MGMT promoters had a longer OS compared to unmethylated MGMT, suggesting a positive predictive value of MGMT methylation status in clinical response to TMZ; moreover, MGMT promoter methylation has been found to be associated with better OS and PFS in IDH mutant GBM patients [61,62]. However, irrespective of MGMT status, patients with TRPML2-positive GSCs showed lower OS and PFS. A strict relationship between EGFRvIII and TRPML2 expression was also observed. Longer OS and PFS were evidenced in EGFRvIII-positive vs. EGFRvIII-negative TRPML2-positive GSC patients, and longer OS values were observed in EGFRvIII-negative vs. EGFRvIII-positive TRPML2-negative GSC patients. Moreover, lower Ki67 levels and negative TRPML2 expression correlate with longer OS and PFS, compared to low Ki67 TRPML2-positive and to high Ki67 TRPML2-positive patients. Finally, in both normal and mutated PTEN, TRPML2-positive vs. TRPML2-negative patients showed shorter OS and PFS. In agreement with our results, EGFRvIII-negative GBM neurosphere cells were more resistant to TMZ than the positive ones. EGFRvIII expression is associated with prolonged OS and PFS of GBM patients treated with surgery and radio/chemotherapy. Depletion of EGFRvIII in recurrent GBMs, as well as differential sensitivity to TMZ in vitro, indicates that the EGFRvIII-negative cells are involved in resistance to radio/chemotherapy [63,64]. Finally, in normal or mutated PTEN expressed GBM patients, Day et al. demonstrated that GBM patients with PTEN mutations exhibited a significantly shorter OS than those without PTEN mutations [65]. A positive correlation between Ki67 staining percentage and OS in GBM patients with IDH-WT has also been reported, with Ki67 staining > 20% predicting poorer PFS [66]. 

In conclusion, data in this study support a relationship between TRPML2 and prognosis in GBM patients. Notably, higher TRPML2 expression levels were found to be strongly related to TMZ resistance in patients’ derived GSCs. Experimental analysis was performed to clarify the role of this channel and we demonstrated that TRPML2 promoted the chemoresistance of GSCs to TMZ affecting PI3K/AKT and JAK/STAT pathways, and drug transporters. We need to acknowledge that there are limitations to our approach. The first is the relatively small number (16) of GSC-derived GBM patients studied whose TRPML2 protein patterns certainly may not recapitulate the full repertoire that exists among GBM patients. Moreover, data reported in GSC#1 and GSC#83 lines represent TMZ sensitivities under cell culture condition, and the in vitro assay may be different from those observed in in vivo clinical conditions. Therefore, the accurate identification of different GSC types in high-grade GBM must be the upcoming task in order to eventually provide significant and personalized therapeutic strategies, instead of applying a standard cure to all patients with GBM. 

## 4. Materials and Methods

### 4.1. Cell Cultures

Neural stem cells (NS/PC#1, NS/PC#2) and GBM stem cell lines (GSC#1, GSC#10, GSC#23, GSC#28, GSC#30, GSC#61, GSC#62, GSC#67, GSC#68, GSC#70, GSC#74, GSC#76, GSC#83, GSC#169, GSC#181 and GSC#195), as previously characterized by [22] were used. The Institute of Neurosurgery, Catholic University School of Medicine in Rome (Italy), isolated these cells from surgical samples of sixteen adult patients with primitive brain tumors with unmethylated and methylated MGMT, that had undergone complete or partial surgical resection from 2006 to 2010 (Table S1). According to the WHO classification, patients were eligible for the study if a diagnosis of GBM was established histologically [67]. The Catholic University School of Medicine’s Ethical Committee obtained informed consent before surgery. Mechanical dissociation of tumor specimens were carried out and cultured in DMEM/F12 serum-free medium containing 2 mM glutamine, 0.6% glucose, 9.6 g/mL putrescine, 6.3 ng/mL progesterone, 5.2 ng/mL sodium selenite, 0.025 mg/mL insulin, and 0.1 mg/mL transferrin sodium salt (Sigma-Aldrich, St. Louis, MO, USA), supplemented with EGF and bFGF before being used to establish GSC cultures. Human GSC lines were authenticated by short tandem repeat (STR) profiling according to the American National Standards Institute/American Type Culture Collection Standard ASN-0002-2011.12 using the Cell Line Integrated Molecular Authentication database (CLIMA), 13 and Cellosaurus STR database (CLASTR) of the Cellosaurus database (ExPASy) at the IRCC Ospedale Policlinico San Martino, Interlab Cell Line Collection (ICLC), Biological Resource Center (CRB-HSM), Genova, Italy [68]. All cell lines were wildtype isocitrate dehydrogenase (IDH) 1/2. 

### 4.2. Chemical and Reagents

The 3-(4,5-dimethylthiazol-2-yl)-2,5-diphenyltetrazolium bromide (MTT) and temozolomide (TMZ) were purchased from Sigma-Aldrich (Milan, Italy). Mouse anti-TRPML2, anti-Bcl2, anti-ADAR1, anti-Survivin and anti-GAPDH antibodies were purchased from Santa Cruz Biotechnology (Santa Cruz, CA, USA). Rabbit anti-STAT3, anti-pSTAT3 Tyr705, anti-pSTAT3 Ser727, anti-Akt, and anti-pAkt Ser473 were purchased from Cell Signaling Technology (Danvers, MA, USA). The following secondary antibodies were used: horseradish peroxidase (HRP)-conjugated sheep anti-mouse IgG and HRP-conjugated donkey anti-rabbit IgG (Cell Signaling Technology).

### 4.3. MTT Assay

Three × 10^4^/mL cells were plated in 96-well plates and treated with different doses of TMZ (125–500 μM), alone or in combination. After that, the samples were incubated for another 3 h with 0.8 mg/mL of MTT. A microtiter plate spectrophotometer (BioTek Instruments, Winooski, VT, USA) was used to measure the color of solutions after the formazan crystals had been dissolved with 100 μL of DMSO per well. Four replicates were used for each treatment. IC_50_ values correspond to the drug concentration that induces 50% of cell growth inhibition compared to control cells. IC_50_ values were calculated using GraphPad Prism^®^ 5.0a (GraphPad Software, San Diego, CA, USA). 

### 4.4. TRPML2 Transfection Models

For silencing experiments, the previously characterized mesenchymal GSC#83 cells (1.2 × 10^5^/mL) [22] were transfected with TRPML2 (siTRPML2, 150 ng) and siCONTROL non-targeting siRNA (siGLO, used as negative control, 150 ng) Flexi Tube siRNA (Qiagen, Milan, Italy), following the HiPerfect transfection reagent transfection protocol (Qiagen). There are no differences between siGLO transfected and untransfected cells. 

For overexpression experiments, the proneural GSC#1 cells (1.5 × 10^5^/mL) [22] were transfected with 10 μL/well of Roti-Fect (Carl Roth GmbH, Karlsruhe, Germany) and 2 μg/well of pCMV3-MCOLN2-t1 (pCMV-TRPML2) (Sino Biological, Wayne, PA, USA) or pCMV3 empty (pCMV) vectors according to the manufacturer’s instructions. There are no differences between pCMV transfected and untransfected cells.

### 4.5. Gene Expression Analysis

Total RNA was extracted with the RNeasy Mini Kit (Qiagen), and cDNA was synthesized using the High-Capacity cDNA Archive Kit (Applied Biosystems, Foster City, PA, USA) according to the manufacturer’s instructions. Quantitative RT-PCR (qRTPCR) was performed by using the IQ5 Multicolor real-time PCR detection system (BioRad, Milan, Italy). The reaction mixture contained the Advanced Universal SYBR Green Supermix (BioRad). Human TRPML2 and GAPDH RT^2^qPCR Primer assay (Qiagen) were used. The PCR parameters were 10 min at 95 °C followed by 40 cycles at 95 °C for 15 s and 60 °C for 40 s. All samples were assayed in triplicate in the same plate. The relative amount of target mRNA was calculated by the 2^−∆∆Ct^ method, using GAPDH as a housekeeping gene.

### 4.6. RT–PCR Profiler Array

Total RNA was extracted from pCMV-TRPML2 and pCMV GSC#1 lines and siTRPML2 and siGLO GSC#83 lines with the RNeasy Mini Kit (Qiagen) and reverse transcribed using the Reaction Ready first strand cDNA kit (Superarray Bioscience Corporation, Frederick, MD, USA). qRT–PCR was performed using the IQ5 Multicolor Real-time PCR detection system (BioRad), the RT^2^ real-time SYBR Green PCR Master Mix and the human ABC transporter plates (Superarray Bioscience Corporation) according to manufacturer’s instructions. The ΔΔCt-based fold change and statistical significance analysis was performed using the Integrated Web-based Software Package for the PCR Array System at the GeneGlobe Data Analysis Center on the Qiagen website.

### 4.7. Western Blot Analysis

GSC#1 and GSC#83 cell lines were lysed in a lysis-buffer containing the protease inhibitor cocktail (Sigma-Aldrich, Milan, Italy). Proteins were separated on 10% SDS polyacrylamide gel, in a Mini-PROTEAN Tetra Cell system (BioRad). Protein transfer from the gel to a nitrocellulose membrane was carried out using Mini Trans-Blot Turbo RTA system (BioRad). Non-specific binding sites were blocked with 5% low-fat dry milk in phosphate-buffered saline 0.1% Tween 20. Membranes were incubated with anti-TRPML2, anti-Bcl2, anti-Survivin or anti-GAPDH (Santa Cruz Biotechnology) primary Abs for 1h at room temperature or with anti-STAT3, anti-pSTAT3 Tyr705, anti-pSTAT3 Ser727, anti-Akt, and anti-pAkt Ser473 primary Abs overnight at 4 °C followed by HRP-conjugated secondary Abs for 1 h at room temperature. The detection was performed using the LiteAblot PLUS (EuroClone, Milan, Italy) kits, and densitometric analysis was carried out by a Chemidoc using the Quantity One software (BioRad). For quantification, GAPDH was used as loading control. One representative out of three independent experiments is shown. 

### 4.8. Protein–Protein Interaction (PPI) Network Analysis

The search tool for retrieval of interacting genes (STRING) (https://string-db.org (accessed on 8 November 2022)) database, based on known and predicted PPIs, was employed to seek potential interactions between the markers [69]. Text mining, experiments, databases, co-expression, species limited to “Homo sapiens”, and an interaction score > 0.7 were considered as active interaction sources and applied to construct the PPI networks. The PPI network was visualized by Cytoscape software version 3.9.1. (https://cytoscape.org/, accessed on 10 November 2022). In the networks, proteins are schematized as nodes and interactions as edges.

### 4.9. Statistical Analysis

All data are expressed as mean ± SD. Student’s *t*-test was used to assess differences between groups. Survival rates were analyzed using the Kaplan–Meier method and differences between groups were compared using the Mantel–Cox tests. All statistical tests are two-tailed. ROC analysis was used to stratify patients according to Ki67 levels (≤20 or >20). A *p* value less than 0.05 was considered statistically significant.

## 5. Conclusions

The GSCs are resistant to conventional therapy, and strategies designed to specifically target them or the pathways involved in stemness characteristics might be useful in the clinic. Therefore, more in-depth studies on resistance mechanisms are needed.

## Data Availability

The data presented in this study are available on request from the corresponding author.

## Supplementary Materials

The following supporting information can be downloaded at: https://www.mdpi.com/article/10.3390/ijms232315356/s1, Figure S1: TRPML2 transfection models in GSC#1 and GSC#83 lines; Table S1: The clinico-pathological characteristics of patients with GBM; Table S2: Kaplan–Meier *p*-values; Table S3: Drug Transporters expression in pCMV-TRPML2 GSC#1 and siTRPML2 GSC#83 lines; Table S4: Kaplan-Meier *p*-values.
