# Supplementary material for: TRPML2 Mucolipin Channels Drive the Response of Glioma Stem Cells to Temozolomide and Affect the Overall Survival in Glioblastoma Patients
Source: Int J Mol Sci. 2022 Dec 5;23(23):15356. doi: 10.3390/ijms232315356 (PMC9738251; doi:10.3390/ijms232315356)
Supplement: Supplementary file 1 [file ijms-23-15356-s001.zip › ijms-1927309-SI.pdf]

Supplementary files

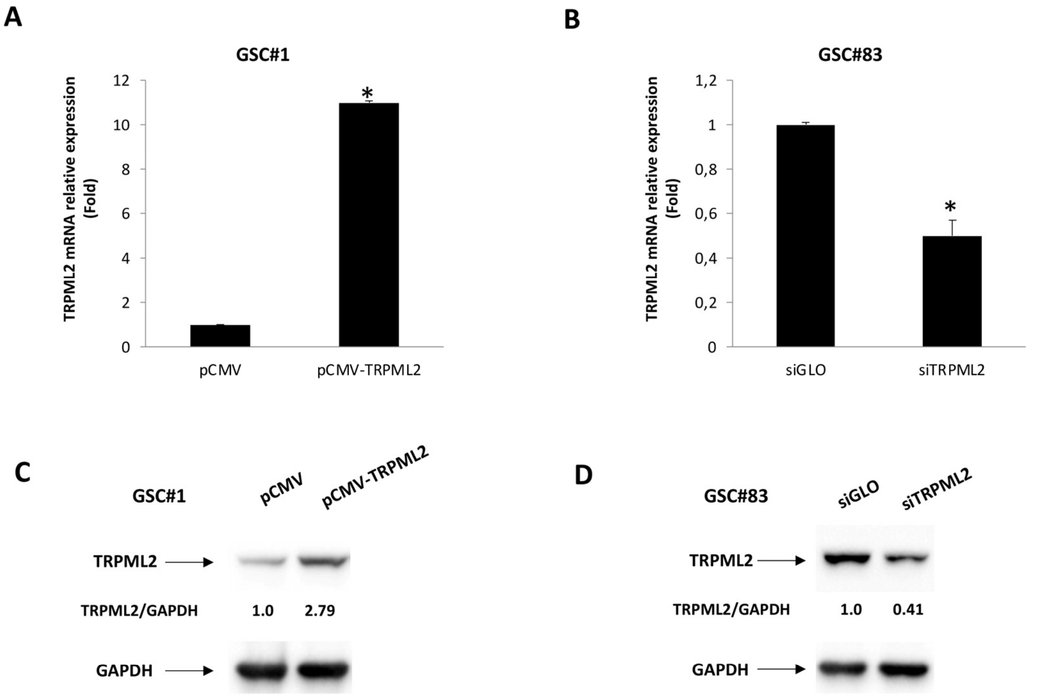

Figure S1. TRPML2 transfection models in GSC#1 and GSC#83 lines. (**A,B**) TRPML2 mRNA levels were evaluated by qRT-PCR after 72 h of pCMV-TRPML2 transfection in GSC#1 (**A**) and siTRPML2 transfection in GSC#83 (**B**). Relative TRPML2 expression, normalized to GAPDH mRNA levels, was calculated using siGLO as calibrator. *$p<0.05$ vs control cells. (**C,D**) Representative immunoblots reflecting TRPML2 protein levels in pCMV and pCMV-TRPML2 GCS#1 (**C**) and in siGLO and siTRPML2 GSC#83 (**D**) after 72 of transfection. Densitometry values were normalized to GAPDH, which was used as loading control.

Table S1. The clinico-pathologic characteristics of patients with GBM.

Variables	Patients (n=16)
Age, years	
range	40-77
median	57.5
Sex	
Male	13/16 (81.25%)
Female	3/16 (18.75%)
Tumor localization	
Temporal	3/16 (18.75%)
Parietal	6/16 (37.50%)
Frontal	6/16 (37.50%)
Occipital	1/16 (6.25%)
Tumor dimention	
ND	4/16 (25.00%)
< 3 cm	3/16 (18.75%)
> 3 cm	9/16 (56.25%)
RxTP preop	
No	15/16 (93.75%)
Yes	6.25 (6.25%)
ASP TOT	
No	3/16 (18.75%)
Yes	13/16 (82.25%)
5-ALA	
No	16/16 (100.00%)
Yes	0/16 (0.00%)
KPSS	
20	1/16 (6.25%)
60-70	8/16 (50.00%)
80-90	7/16 (43.75%)
Recidive	
No	15/16 (93.75%)
Yes	1/16 (6.25%)
MGMT promoter status	
Unmethylated	10/16 (62.25%)
Methylated	6/16 (37.75%)
EGFRvIII	
Negative	9/16 (56.25%)
Positive	7/16 (43.75%)
PTEN	
ND	1/16 (6.25%)
Normal	11/16 (68.75%)
Hypophosphorilated	4/16 (25.00%)
Ki67	
≤ 20	10/16 (62.25%)
> 20	6/16 (37.50%)

Table S2. Drug Transporters expression in GSC#1 and GSC#83 lines.

	GSC#1	GSC#83		GSC#1	GSC#83
**Target genes**	**mRNA relative expression**	**mRNA relative expression**	**Target genes**	**mRNA relative expression**	**mRNA relative expression**
**ABCA1**	0,0000 ± 0,0000	0,0226 ± 0,0060	**SLC19A1**	0,0065 ± 0,0026	0,0133 ± 0,0038
**ABCA12**	0,0011 ± 0,0038	0,0045 ± 0,0013	**SLC19A2**	0,0138 ± 0,0075	0,0185 ± 0,0053
**ABCA13**	0,0000 ± 0,0000	0,0036 ± 0,0010	**SLC19A3**	0,0040 ± 0,0018	0,0037 ± 0,0011
**ABCA2**	0,0184 ± 0,0630	0,0402 ± 0,0014	**SLC22A1**	0,0000 ± 0,0000	0,0022 ± 0,0006
**ABCA3**	0,0306 ± 0,1051	0,0382 ± 0,0019	**SLC22A2**	0,0000 ± 0,0000	0,0000 ± 0,0000
**ABCA4**	0,0046 ± 0,0159	0,0012 ± 0,0003	**SLC22A3**	0,0000 ± 0,0000	0,0064 ± 0,0018
**ABCA5**	0,0122± 0,0418	0,0168 ± 0,0048	**SLC22A6**	0,0000 ± 0,0000	0,0000 ± 0,0000
**ABCA9**	0,0000 ± 0,0000	0,0077 ± 0,0012	**SLC22A7**	0,0003 ± 0,0001	0,0000 ± 0,0000
**ABCB1**	0,0000 ± 0,0000	0,0000 ± 0,0000	**SLC22A8**	0,0003 ± 0,0002	0,0024 ± 0,0007
**ABCB11**	0,0000 ± 0,0000	0,0000 ± 0,0000	**SLC22A9**	0,0000 ± 0,0000	0,0000 ± 0,0000
**ABCB4**	0,0008 ± 0,0029	0,0027 ± 0,0008	**SLC25A13**	0,0192 ± 0,0058	0,0449 ± 0,0028
**ABCB5**	0,0000 ± 0,0000	0,0000 ± 0,0000	**SLC28A1**	0,0006 ± 0,0002	0,0013 ± 0,0004
**ABCB6**	0,0320 ± 0,1097	0,0505 ± 0,0044	**SLC28A2**	0,0011 ± 0,0004	0,0015 ± 0,0004
**ABCC1**	0,0093 ± 0,0321	0,0310 ± 0,0088	**SLC28A3**	0,0011 ± 0,0003	0,0016 ± 0,0004
**ABCC10**	0,0042 ± 0,0146	0,0179 ± 0,0051	**SLC29A1**	0,0220 ± 0,0055	0,0399 ± 0,0013
**ABCC11**	0,0010 ± 0,0037	0,0016 ± 0,0005	**SLC29A2**	0,0127 ± 0,0037	0,0102 ± 0,0029
**ABCC12**	0,0000 ± 0,0000	0,0000 ± 0,0000	**SLC2A1**	0,3037 ± 0,0147	0,1110 ± 0,0015
**ABCC2**	0,0003 ± 0,0002	0,0096 ± 0,0007	**SLC2A2**	0,0000 ± 0,0000	0,0000 ± 0,0000
**ABCC3**	0,0077 ± 0,0065	0,0360 ± 0,0012	**SLC2A3**	0,1508 ± 0,0068	0,0235 ± 0,0067
**ABCC4**	0,0138 ± 0,0073	0,0241 ± 0,0069	**SLC31A1**	0,0164 ± 0,0064	0,0714 ± 0,0003
**ABCC5**	0,0100 ± 0,0045	0,0297 ± 0,0085	**SLC38A2**	0,0782 ± 0,0080	0,2731 ± 0,0377
**ABCD1**	0,0096 ± 0,0032	0,0333 ± 0,0095	**SLC38A5**	0,0019 ± 0,0008	0,0026 ± 0,0007
**ABCD3**	0,0474 ± 0,0026	0,0679 ± 0,0193	**SLC3A1**	0,0027 ± 0,0002	0,0031 ± 0,0008
**ABCD4**	0,0191 ± 0,0055	0,0363 ± 0,0003	**SLC3A2**	0,1294 ± 0,0133	0,2270 ± 0,0446
**ABCF1**	0,0224 ± 0,0067	0,0681 ± 0,0194	**SLC5A1**	0,0010 ± 0,0005	0,0000 ± 0,0000
**ABCG2**	0,0037 ± 0,0019	0,0059 ± 0,0017	**SLC5A4**	0,0000 ± 0,0000	0,0018 ± 0,0005
**ABCG8**	0,0000 ± 0,0000	0,0000 ± 0,0000	**SLC7A11**	0,0039 ± 0,0005	0,0329 ± 0,0093
**AQP1**	0,0346 ± 0,0087	0,1644 ± 0,0468	**SLC7A5**	0,1054 ± 0,0113	0,2235 ± 0,0136
**AQP7**	0,0000 ± 0,0000	0,0014 ± 0,0004	**SLC7A6**	0,0080 ± 0,0007	0,0324 ± 0,0092
**AQP9**	0,0000 ± 0,0000	0,0014 ± 0,0004	**SLC7A7**	0,0027 ± 0,0003	0,0052 ± 0,0005
**ATP6VOC**	0,0645 ± 0,0010	0,1268 ± 0,0361	**SLC7A8**	0,0104 ± 0,0009	0,0066 ± 0,0019
**ATP7A**	0,0067 ± 0,0031	0,021 ± 0,0060	**SLC7A9**	0,0000 ± 0,0000	0,0008 ± 0,0002
**ATP7B**	0,0037 ± 0,0018	0,002 ± 0,0006	**SLC01A2**	0,0000 ± 0,0000	0,0000 ± 0,0000
**MVP**	0,0157 ± 0,0038	0,1101 ± 0,0313	**SLC01B1**	0,0005 ± 0,0002	0,0008 ± 0,0002
**SLC10A1**	0,0005 ± 0,0018	0,0009 ± 0,0003	**SLC01B3**	0,0000 ± 0,0000	0,0000 ± 0,0000
**SLC10A2**	0,0000 ± 0,0000	0,0000 ± 0,0000	**SLC02A1**	0,0009 ± 0,0001	0,0174 ± 0,0049
**SLC15A1**	0,0000 ± 0,0000	0,0007 ± 0,0002	**SLC02B1**	0,0000 ± 0,0000	0,0005 ± 0,0001
**SLC15A2**	0,0061 ± 0,0009	0,0017 ± 0,0005	**SLC03A1**	0,0004 ± 0,0001	0,0671 ± 0,0090
**SLC16A1**	0,0791 ± 0,0010	0,1034 ± 0,0294	**SLC04A1**	0,0449 ± 0,0039	0,0904 ± 0,0257
**SLC16A2**	0,0059 ± 0,0003	0,0265 ± 0,0075	**VDAC1**	0,0445 ± 0,0027	0,1383 ± 0,0393
**SLC16A3**	0,0124 ± 0,0025	0,0405 ± 0,0015	**VDAC2**	0,0911 ± 0,0024	0,1433 ± 0,0408

Human Drug Transporters RT profiler PCR array in mRNA samples extracted from GSC#1 and GSC#83. The expression levels were normalized to the average Ct value of ACTB and calculated by the ΔΔCt method. ABCA1, ATP-binding cassette, sub-family A (ABC1), member 1 (ABCA1); ABC1 member 12 (ABCA12); ABC1 member 13 (ABCA13); ABC1 member 2 (ABCA2); ABC1, member 3 (ABCA3); ABC1 member 4 (ABCA4); ABC1 member 5 (ABCA5); ABC1 member 9 (ABCA9); ATP-binding cassette, sub-family B (MDR/TAP), member 1 (ABCB1); MDR/TAP member 11 (ABCB11); MDR/TAP member 4 (ABCB4); MDR/TAP member 5 (ABCB5); MDR/TAP, member 6 (ABCB6); (CFTR/MRP), member 1 (ABCC1); CFTR/MRP member 10 (ABCC10); CFTR/MRP member 11 (ABCC11); CFTR/MRP member 12 (ABCC12); CFTR/MRP member 2

(ABCC2); CFTR/MRP member 3 (ABCC3); CFTR/MRP member 4 (ABCC4); CFTR/MRP member 5 (ABCC5); ATP-binding cassette, sub-family D (ALD), member 1 (ABCD1); ALD member 3 (ABCD3); ALD member 4 (ABCD4); ATP-binding cassette, sub-family F (GCN20), member 1 (ABCF1); ATP-binding cassette, sub-family G (WHITE), member 2 (ABCG2); WHITE member 8 (ABCG8); Aquaporin 1 (AQP1); Aquaporin 7 (AQP7); Aquaporin 9 (AQP9); ATPase, H+ transporting, lysosomal 16kDa, V0 subunit c (ATP6V0C); ATPase, Cu++ transporting, alpha polypeptide (ATP7A); ATPase, Cu++ transporting, beta polypeptide (ATP7B); Major vault protein (MVP); Solute carrier family 10 (sodium/bile acid cotransporter family), member 1 (SLC10A1); Solute carrier family 10 (sodium/bile acid cotransporter family), member 2 (SLC10A2); Solute carrier family 15 (oligopeptide transporter), member 1 (SLC15A1); Solute carrier family 15 (H+/peptide transporter), member 2 (SLC15A2); Solute carrier family 16, member 1 (monocarboxylic acid transporter 1) (SLC16A1); Solute carrier family 16, member 2 (monocarboxylic acid transporter 8)(SLC16A2); Solute carrier family 16, member 3 (monocarboxylic acid transporter 4) (SLC16A3); Solute carrier family 19 (folate transporter), member 1 (SLC19A1); Solute carrier family 19 (thiamine transporter), member 2 (SLC19A2); Solute carrier family 19, member 3 (SLC19A3); Solute carrier family 22 (organic cation transporter), member 1 (SLC22A1); Solute carrier family 22 (organic cation transporter), member 2 (SLC22A2); Solute carrier family 22 (extraneuronal monoamine transporter), member 3 (SLC22A3); Solute carrier family 22 (organic anion transporter), member 6 (SLC22A6);Solute carrier family 22 (organic anion transporter), member 7 (SLC22A7); Solute carrier family 22 (organic anion transporter), member 8 (SLC22A8); Solute carrier family 22 (organic anion transporter), member 9 (SLC22A9); Solute carrier family 25, member 13 (citrin) (SLC25A13);Solute carrier family 28 (sodium-coupled nucleoside transporter), member 1 (SLC28A1); Solute carrier family 28 (sodium-coupled nucleoside transporter), member 2 (SLC28A2); Solute carrier family 28 (sodium-coupled nucleoside transporter), member 3 (SLC28A3);Solute carrier family 29 (nucleoside transporters), member 1 (SLC29A1); Solute carrier family 29 (nucleoside transporters), member 2 (SLC29A2); Solute carrier family 2 (facilitated glucose transporter), member 1 (SLC2A1); Solute carrier family 2 (facilitated glucose transporter), member 2 (SLC2A2); Solute carrier family 2 (facilitated glucose transporter), member 3 (SLC2A3); Solute carrier family 31 (copper transporters), member 1 (SLC31A1); Solute carrier family 38, member 2 (SLC38A2); Solute carrier family 38, member 5 (SLC38A5); Solute carrier family 3 (cystine, dibasic and neutral amino acid transporters, activator of cystine, dibasic and neutral amino acid transport), member 1 (SLC3A1); Solute carrier family 3 (activators of dibasic and neutral amino acid transport), member 2 (SLC3A2); Solute carrier family 5 (sodium/glucose cotransporter), member 1 (SLC5A1); Solute carrier family 5 (low affinity glucose cotransporter), member 4 (SLC5A4); Solute carrier family 7 (anionic amino acid transporter light chain, xc- system), member 11 (SLC7A11); Solute carrier family 7 (amino acid transporter light chain, L system), member 5 (SLC7A5); Solute carrier family 7 (amino acid transporter light chain, y+L system), member 6 (SLC7A6); Solute carrier family 7 (amino acid transporter light chain, y+L system), member 7 (SLC7A7); Solute carrier family 7 (amino acid transporter light chain, L system), member 8 (SLC7A8); Solute carrier family 7 (glycoprotein-associated amino acid transporter light chain, bo,+ system), member 9 (SLC7A9); Solute carrier organic anion transporter family, member 1A2 (SLCO1A2); Solute carrier organic anion transporter family, member 1B1 (SLCO1B1); Solute carrier organic anion transporter family, member 1B3 (SLCO1B3); Solute carrier organic anion transporter family, member 2A1 (SLCO2A1); Solute carrier organic anion transporter family, member 2B1 (SLCO2B1); Solute carrier organic anion transporter family, member 3A1 (SLCO3A1); Solute carrier organic anion transporter family, member 4A1 (SLCO4A1); Voltage-dependent anion channel 1 (VDAC1); Voltage-dependent anion channel 2 (VDAC2).

Table S3. Drug Transporters expression in pCMV-TRPML2 GSC#1 and siTRPML2 GSC#83 lines.

	pCMV-TRPML2 GSC#1	siTRPML2 GSC#83
**Target genes**	**Fold expression**	**Fold expression**
ABCA1	Induced	2.16 ± 0.41
ABCA12	3.72 ± 0.70	
ABCB1		Induced
ABCB4	Silenced	0.27 ± 0.05
ABCB5		Induced
ABCB11	Silenced	Induced
ABCC2	8.10 ± 1.51	
ABCC3	0.64 ± 0.03	1.61 ± 0.23
ABCC11	Silenced	
ABCD1	0.96 ± 0.17	2.10 ± 0.39
ABCD4	2.20 ± 0.41	
ABCG2	0.95 ± 0.13	1.34 ± 0.12
AQP1	0.85 ± 0.19	1.15 ± 0.12
AQP7		2.16 ± 0.40
AQP9	Induced	
ATP6VOC	0.85 ± 0.21	1.08 ± 0.31
ATP7B	0,91 ± 0.07	3.03 ± 0.56
SLC10A1	2.89 ± 0.53	1.3 ± 0.41
SLC15A1		Silenced
SLC15A2		Silenced
SLC16A1	0.87 ± 0.09	1.59 ± 0.21
SLC16A2	0.55 ± 0.10	
SLC22A1		2.20 ± 0.41
SLC22A2	Induced	
SLC22A7	5.78 ± 1.08	
SLC22A8	Silenced	Silenced
SLC25A13	0.85 ± 0.11	
SLC28A1	2.42 ± 0.45	0.91 ± 0.09
SLC28A2	4.02 ± 0.75	0.79 ± 0.12
SLC29A1	0.89 ± 0.05	1.64 ± 0.25
SLC29A2	0.91 ± 0.07	1.43 ± 0.17
SLC28A3	2.05 ± 0.38	
SLC38A5	2.55 ± 0.48	
SLC2A1	0.65 ± 0.12	1.47 ± 0.12
SLC2A2		Induced
SLC3A1	0.84 ± 0.08	1.71 ± 0.33
SLC5A1	Silenced	
SLC5A4	Induced	2.60 ± 0.49
SLC7A7	2.40 ± 0.36	
SLC7A8	0.87 ± 0.07	1.54 ± 0.23
SLC7A9	Induced	
SLCO1B1	2.42 ± 0.36	2.81 ± 0.52
SLCO2A1	0.85 ± 0.08	1.68 ± 0.26
SLCO2B1	2.10 ± 0.39	
SLCO3A1	Silenced	1.34 ± 0.11

Human Drug Transporters RT profiler PCR array in mRNA samples extracted from GSC#1. The expression levels were normalized to the average Ct value of two housekeeping genes (ACTB and RPLP0) and calculated by the ΔΔCt method. Values represent fold differences of individual gene expression in pCMV-TRPML2 compared to pCMV GSC#1 and siTRPML2 compared to siGLO GSC#83 cells.

ABCA1, ATP-binding cassette, sub-family A (ABC1), member 1 (ABCA1); ABC1 member 12 (ABCA12); ATP-binding cassette, sub-family B (MDR/TAP), member 1 (ABCB1); ATP-binding cassette, sub-family B (MDR/TAP), member 11 (ABCB11); MDR/TAP member 4 (ABCB4); MDR/TAP member 5 (ABCB5); CFTR/MRP member 2

(ABCC2); CFTR/MRP member 3 (ABCC3); CFTR/MRP member 11 (ABCC11); ALD member 4 (ABCD4); ATP-binding cassette, sub-family G (WHITE), member 2 (ABCG2); Aquaporin 1 (AQP1); Aquaporin 7 (AQP7); Aquaporin 9 (AQP9); ATPase, H+ transporting, lysosomal 16kDa, V0 subunit c (ATP6V0C); ATPase, Cu++ transporting, beta polypeptide (ATP7B); Solute carrier family 10 (sodium/bile acid cotransporter family), member 1 (SLC10A1); Solute carrier family 15 (oligopeptide transporter), member 1 (SLC15A1); Solute carrier family 15 (H+/peptide transporter), member 2 (SLC15A2); Solute carrier family 16, member 1 (monocarboxylic acid transporter 1) (SLC16A1); Solute carrier family 16, member 2 (monocarboxylic acid transporter 8)(SLC16A2); Solute carrier family 22 (organic cation transporter), member 1 (SLC22A1); Solute carrier family 22 (organic cation transporter), member 2 (SLC22A2); Solute carrier family 22 (organic anion transporter), member 7 (SLC22A7); Solute carrier family 22 (organic anion transporter), member 8 (SLC22A8); Solute carrier family 28 (sodium-coupled nucleoside transporter), member 1 (SLC28A1); Solute carrier family 28 (sodium-coupled nucleoside transporter), member 2 (SLC28A2); Solute carrier family 28 (sodium-coupled nucleoside transporter), member 3 (SLC28A3);Solute carrier family 29 (nucleoside transporters), member 1 (SLC29A1); Solute carrier family 29 (nucleoside transporters), member 2 (SLC29A2); Solute carrier family 2 (facilitated glucose transporter), member 1 (SLC2A1); Solute carrier family 2 (facilitated glucose transporter), member 2 (SLC2A2); Solute carrier family 31 (copper transporters), member 1 (SLC31A1); Solute carrier family 5 (sodium/glucose cotransporter), member 1 (SLC5A1); Solute carrier family 5 (low affinity glucose cotransporter), member 4 (SLC5A4); Solute carrier family 7 (amino acid transporter light chain, y+L system), member 7 (SLC7A7); Solute carrier family 7 (amino acid transporter light chain, L system), member 8 (SLC7A8); Solute carrier family 7 (glycoprotein-associated amino acid transporter light chain, bo,+ system), member 9 (SLC7A9); Solute carrier organic anion transporter family, member 1B1 (SLCO1B1); Solute carrier organic anion transporter family, member 2A1 (SLCO2A1); Solute carrier organic anion transporter family, member 2B1 (SLCO2B1); Solute carrier organic anion transporter family, member 3A1 (SLCO3A1).

Table S4. Kaplan-Meier p-values.

OS, p value	Methylated MGMT TRPML2 negative	Methylated MGMT TRPML2 positive	Unmethylated MGMT TRPML2 negative	Unmethylated MGMT TRPML2 positive
**Methylated MGMT TRPML2 negative**		0.0295 *	0.9876	0.0094 *
**Methylated MGMT TRPML2 positive**	0.0295 *		0.0295 *	0.6392
**Unmethylated MGMT TRPML2 negative**	0.9876	0.0295 *		0.0094 *
**Unmethylated MGMT TRPML2 positive**	0.0094 *	0.6392	0.0094 *	

PFS, p value	Methylated MGMT TRPML2 negative	Methylated MGMT TRPML2 positive	Unmethylated MGMT TRPML2 negative	Unmethylated MGMT TRPML2 positive
**Methylated MGMT TRPML2 negative**		0.0295 *	0.8292	0.0168 *
**Methylated MGMT TRPML2 positive**	0.0295 *		0.2254	0.1048
**Unmethylated MGMT TRPML2 negative**	0.8292	0.2254		0.0094 *
**Unmethylated MGMT TRPML2 positive**	0.0168 *	0.1048	0.0094 *	

OS, p value	EGFRvIII negative TRPML2 negative	EGFRvIII negative TRPML2 positive	EGFRvIII positive TRPML2 negative	EGFRvIII positive TRPML2 positive
EGFRvIII negative TRPML2 negative		0.0027 *	0.0253 *	0.0023 *
EGFRvIII negative TRPML2 positive	0.0027 *		0.1161	0.0013 *
EGFRvIII positive TRPML2 negative	0.0253 *	0.1161		0.0928
EGFRvIII positive TRPML2 positive	0.0023 *	0.0013 *	0.0928	

PFS, p value	EGFRvIII negative TRPML2 negative	EGFRvIII negative TRPML2 positive	EGFRvIII positive TRPML2 negative	EGFRvIII positive TRPML2 positive
EGFRvIII negative TRPML2 negative		0.0047 *	0.7046	0.0109 *
EGFRvIII negative TRPML2 positive	0.0047 *		0.0455 *	0.0027 *
EGFRvIII positive TRPML2 negative	0.7046	0.0455 *		0.0895
EGFRvIII positive TRPML2 positive	0.0109 *	0.0027 *	0.0895	

OS, p value	Ki67 ≤ 20 TRPML2 negative	Ki67 ≤ 20 TRPML2 positive	Ki67 > 20 TRPML2 positive
Ki67 ≤ 20 TRPML2 negative		0.0013 *	0.0007 *
Ki67 ≤ 20 TRPML2 positive	0.0013 *		0.7824
Ki67 > 20 TRPML2 positive	0.0007 *	0.7824	

PFS, p value	Ki67 ≤ 20 TRPML2 negative	Ki67 ≤ 20 TRPML2 positive	Ki67 > 20 TRPML2 positive
Ki67 ≤ 20 TRPML2 negative		0.0140 *	0.0009 *
Ki67 ≤ 20 TRPML2 positive	0.0140 *		0.2449
Ki67 > 20 TRPML2 positive	0.0009 *	0.2449	

OS, p value	Normal PTEN TRPML2 negative	Normal PTEN TRPML2 positive	Hypo PTEN TRPML2 negative	Hypo PTEN TRPML2 positive
**Normal PTEN TRPML2 negative**		0.0089 *	0.2072	0.0389 *
**Normal PTEN TRPML2 positive**	0.0089 *		0.0253 *	0.0742
**Hypo PTEN TRPML2 negative**	0.2072	0.0253 *		0.0896
**Hypo PTEN TRPML2 positive**	0.0389 *	0.0742	0.0896	

PFS, p value	Normal PTEN TRPML2 negative	Normal PTEN TRPML2 positive	Hypo PTEN TRPML2 negative	Hypo PTEN TRPML2 positive
**Normal PTEN TRPML2 negative**		0.043 *	0.8864	0.0455 *
**Normal PTEN TRPML2 positive**	0.043 *		0.0228 *	0.0662
**Hypo PTEN TRPML2 negative**	0.8864	0.0228 *		0.0833
**Hypo PTEN TRPML2 positive**	0.0455 *	0.0662	0.0833	
